# Factors That Affect the Formation of Chromosomal Translocations in Cells

**DOI:** 10.3390/cancers14205110

**Published:** 2022-10-18

**Authors:** Reynand Jay Canoy, Anna Shmakova, Anna Karpukhina, Mikhail Shepelev, Diego Germini, Yegor Vassetzky

**Affiliations:** 1 UMR 9018, CNRS, Université Paris Saclay, Institut Gustave Roussy, F-94805 Villejuif, France; 2 Institute of Human Genetics, National Institutes of Health, University of the Philippines Manila, Manila 1000, Philippines; 3 Scilore Asia-Pacific Corporation, Ayala-Alabang, Muntinlupa City 1780, Philippines; 4 Koltzov Institute of Developmental Biology, 117334 Moscow, Russia; 5 Institute of Gene Biology, 117334 Moscow, Russia

**Keywords:** chromosomal translocation, cancer, translocation formation, spatial proximity

## Abstract

**Simple Summary:**

Chromosomal translocations are products of the erroneous repair of DNA double-strand breaks that result in the illegitimate joining of the two broken chromosomal ends from non-homologous chromosomes. Chromosomal translocations have been linked to aneuploidy, infertility, mental retardation, cancer and other diseases. Here we discuss how chromosomal translocations are formed and explore how different cellular factors contribute to their formation.

**Abstract:**

Chromosomal translocations are products of the illegitimate repair of DNA double-strand breaks (DSBs). Their formation can bring about significant structural and molecular changes in the cell that can be physiologically and pathologically relevant. The induced changes may lead to serious and life-threatening diseases such as cancer. As a growing body of evidence suggests, the formation of chromosomal translocation is not only affected by the mere close spatial proximity of gene loci as potential translocation partners. Several factors may affect formation of chromosomal translocations, including chromatin motion to the potential sources of DSBs in the cell. While these can be apparently random events, certain chromosomal translocations appear to be cell-type-specific. In this review, we discuss how chromosomal translocations are formed and explore how different cellular factors contribute to their formation.

## 1. Introduction

Chromosomal translocations are the products of an illegitimate proximity-based repair of DNA double-strand breaks (DSBs), where the proximity of DNA ends from different non-homologous chromosomes can result in their erroneous joining. The appearance of a chromosomal translocation results in substantial structural and molecular alterations in the cell and may have perilous pathological consequences. The mechanisms of chromosomal translocation generation, as well as their contribution to human diseases, were extensively reviewed before (e.g., [1,2,3,4]). Still, it is currently unknown why certain chromosomal translocations are observed in specific cell types. In the current review we aim to summarize cellular factors that contribute to the formation of chromosomal translocations and that, among other things, could explain the specificity of the occurrence of certain chromosomal translocations.

### 1.1. History of Chromosomal Translocations in Cancer

The link between an aberrant number of chromosomes and cancer was first described by Theodor Boveri, who postulated that “growth stimulatory chromosomes” triggered the unlimited growth of tumor cells [5,6]. Then, in 1960, the Philadelphia chromosome, which appeared to be smaller than chromosomes 21 and 22, was described in patients with chronic myeloid leukemia (CML) [7]. However, it was not until 1973 that Janet Rowley showed that this chromosome is the truncated derivative of chromosome 22 fused to the fragment of chromosome 9 [8,9,10]. This report was the first to demonstrate that the novel chromosomes, which were usually reported to be present in tumor cells, were actually the products of chromosomal translocations.

### 1.2. Types of Chromosomal Translocations

**Figure 1:**
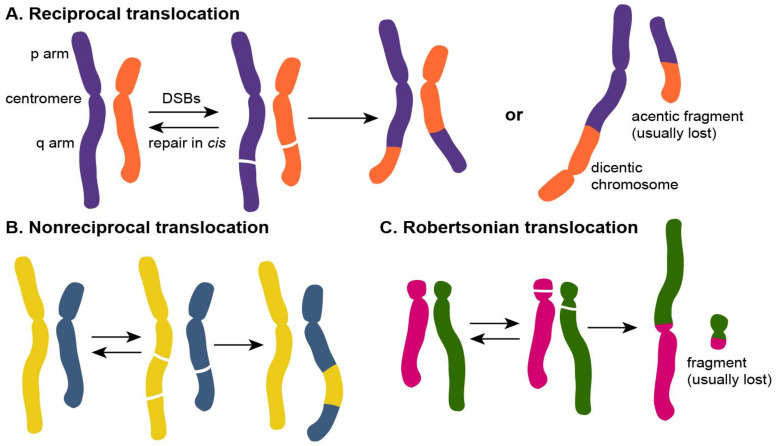
Types of chromosomal translocations. The illegitimate repair of the two DSBs in two non-homologous chromosomes can produce (**A**) reciprocal translocations if the exchange between two translocating chromosomes is bidirectional or (**B**) non-reciprocal translocations if the exchange is unidirectional. (**C**) Robertsonian translocations occur between two acrocentric chromosomes that are fused at the centromere.

Chromosomal translocations are generated when non-homologous chromosomes exchange their parts with each other. A few types of chromosomal translocations have been described [11,12]. Chromosomal translocations are considered reciprocal (Figure 1A) if the exchange between chromosomes is bidirectional. Non-reciprocal chromosomal translocations result from the unidirectional transfer of one chromosomal part to another chromosome (Figure 1B). Chromosomal translocations can also be classified as balanced (no net loss or gain of genomic material) or unbalanced if genetic material is lost or amplified. In reciprocal translocations, one of the three sets of possible derivative chromosomes can be produced [13]: 

(1) Two chromosomes produce monocentric derivative chromosomes with one centromere (Figure 1A); 

(2) Two chromosomes produce a derivative chromosome with two centromeres (dicentric) and a derivative chromosome without a centromere (acentric) (Figure 1A); 

(3) Two acrocentric chromosomes fuse at the centromere (Figure 1C) (Robertsonian translocation; [14,15]). The remnants of the short p arms of the two chromosomes also fuse and the small reciprocal product are usually lost, which may not be deleterious for the cell [16].

The dicentric and acentric chromosomes are often lost after several rounds of cell divisions as they are either lethal or unstable [11], while Robertsonian translocations may persist in meiosis and even produce gametes [12].

### 1.3. Chromosomal Translocations Affect the Normal Cell Functions

**Figure 2:**
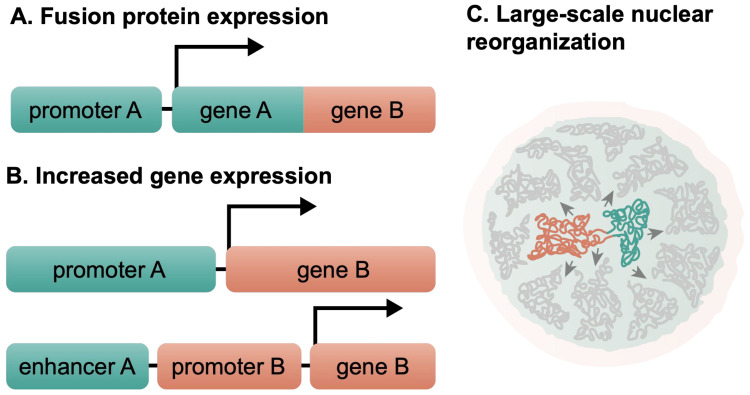
Molecular and functional consequences of chromosomal translocations. Chromosomal translocations can bring about the formation of fusion protein (**A**) or the (**B**) aberrant expression of an otherwise normal gene as well as (**C**) large-scale changes in the nuclear organization have also been attributed to chromosomal translocations.

The mere rearrangement of chromosome parts can cause significant consequences in the cell. Chromosomal translocations have been linked to aneuploidy, infertility, mental retardation, cancer, and other diseases [17,18,19,20,21,22]. Chromosomal translocations may result in the formation of fusion proteins (Figure 2A) with unique or atypical functions or activity, as in the case of the BCR-ABL fusion protein in CML [23,24,25]. In addition, they can result in the aberrant expression (upregulation or downregulation) of an otherwise normal gene if it is positioned next to the new cis-regulatory elements (Figure 2B). Usually these aberrantly expressed genes are proto-oncogenes that are controlled by a potent gene promoter or enhancer after the translocation. Either of these two can significantly affect the cell and can lead to its transformation and malignancy. This is exemplified by Burkitt’s lymphoma, where the *MYC* gene on chromosome 8 is translocated next to the *IGH* locus on chromosome 14; this triggers its overexpression [26,27,28,29,30]. Recently, large-scale changes in the nuclear organization have also been attributed to chromosomal translocations, further expanding our understanding of the consequences of chromosomal translocations for the cell [11,31,32,33,34,35,36].

Chromosomal translocations are characteristic for many types of cancer [37,38], including carcinomas [39,40], lymphomas, leukemias [19,37], and soft tissue sarcomas [20,41,42]. The Mitelman Database of Chromosome Aberrations and Gene Fusions in Cancer (https://mitelmandatabase.isb-cgc.org/ (accessed on 27 September 2022)) contains exhaustive data related to chromosomal aberrations, including translocations and cancer [43].

## 2. Sources of DSBs In Vivo and Their Experimental Modeling for Studying Translocations

### 2.1. Sources of DSBs

The sources of DSBs can be grouped into extrinsic and intrinsic ones. The former include environmental factors such as the ionizing radiation of X-rays and alpha particles [44,45,46,47,48,49], UV light, or exposure to genotoxic chemical agents (e.g., chemotherapy). Cellular activities that involve DNA-cutting enzymes or produce nonspecific DNA damage, are the potential intrinsic sources of DSBs. In meiotic recombination, DSBs are induced mainly by SPO11 [50,51] during the segregation of chromosomes to convert the cell from diploid to haploid [52,53]. DSBs are consequently produced in lymphocytes during the V(D)J recombination in maturing B and T cells [54,55,56]. Furthermore, DSBs are also formed during the somatic hypermutation (SHM) and the immunoglobulin class switching or class switch recombination (CSR) in mature B cells [57,58]. The breaks in V(D)J recombination are initiated by the recombination-activating gene (RAG) 1/2 complex [59], while the breaks in SHM and CSR are initiated by the activation-induced deaminase (AID) [60]. Although AID has a special affinity for immunoglobulin genes, it is able to target a large number of other genes (~25% of all expressed genes in B cells) [61]. This programmed DNA damage is considered as a major contributing factor to the susceptibility of B-cells to chromosomal translocations [1]. On the other hand, there are cellular activities that unintentionally produce DSBs such as gene transcription [62,63,64,65,66,67], DNA replication [68,69] and oxidative metabolism [70,71].

Apparently, different sources of DSB and the chromosome regions targeted by these DSBs differently affect the formation of chromosomal translocations; different sources of DSBs might also determine which factors influence the formation of translocations and thus, translocation specificity, which is discussed below. For instance, CRISPR/Cas9 mostly induce DSBs independently of the chromatin context, which makes the generation of CRISPR/Cas9-induced translocations dependent on the spatial proximity of DNA ends and the choice of DSB repair pathway [72,73,74]. This should also be taken into account when modeling chromosomal translocations by various methods of DSB induction. 

### 2.2. Experimental Models of Chromosomal Translocations

**Figure 3:**
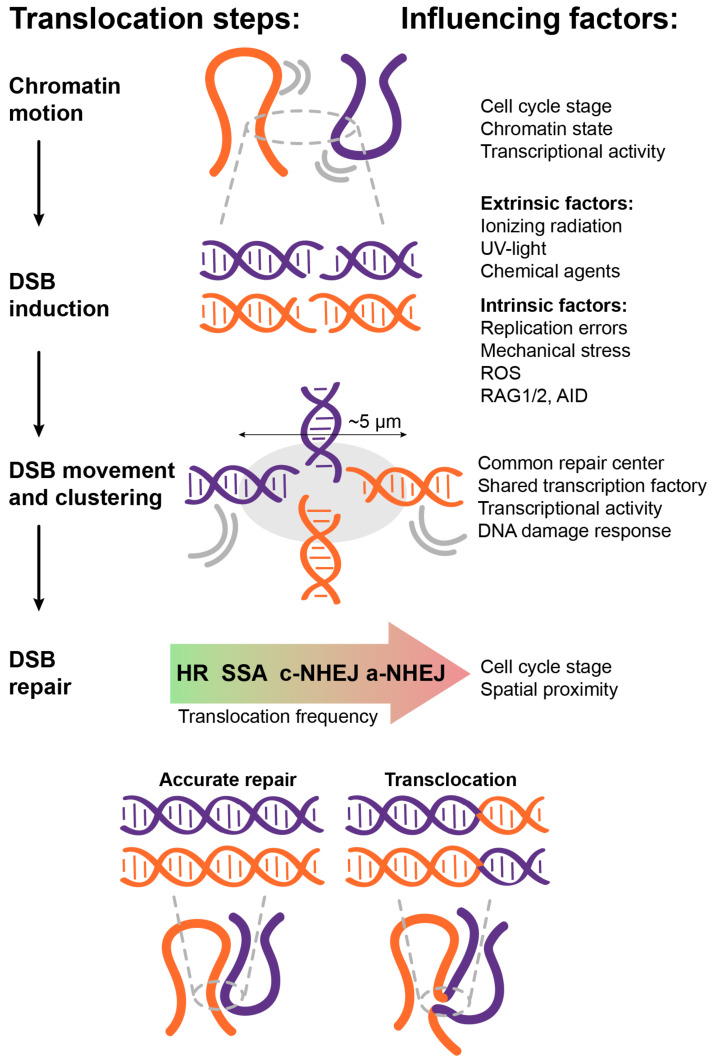
Different factors affect the formation of chromosomal translocations. These cellular factors are not mutually exclusive and can influence each other to affect the multiple stages of chromosomal translocation formation.

Translocations can be induced either randomly (e.g., by treatment with γ-rays or H_2_O_2_) or specifically (e.g., by engineered programmed nucleases) [75]. The advent of engineered nucleases provided a new opportunity to precisely study translocations by introducing DSBs in the regions of interest. Specific chromosomal translocations can now be generated from precisely induced DSBs using programmed nucleases such as zinc finger nucleases (ZFN), transcription activator-like effector nucleases (TALEN), and clustered regularly interspaced short palindromic repeats (CRISPR) [76,77,78,79,80,81,82,83,84]. A more sophisticated way to induce chromosomal translocations in cells would be to use proteins that naturally induce DNA damage and are known to be implicated in chromosomal translocations, reviewed in [85,86]. In B cells, the chromosome breaks are usually caused by either AID, involved in class switch recombination and somatic hypermutation, or RAG endonuclease, essential for V(D)J recombination together with its RAG2 cofactor. Consequently, physiological or aberrant activation of these genes may induce DSBs and chromosomal translocations. These translocations can then be identified by next-generation sequencing [87,88]. A subset of chromosomal translocations in hematological and solid tumors are driven by DNA topoisomerse II (TOP2) [89]. TOP2 poisons, such as the anticancer agent etoposide, which trap DNA-TOP2 complexes, can be used to induce and identify chromosomal translocations [90]. The factors favoring chromosomal translocations (Figure 3) identified in these and other studies are considered below. 

## 3. How Do the Different Factors Affect the Formation of Chromosomal Translocations?

### 3.1. Spatial Proximity

The erroneous repair of DSBs on different non-homologous chromosomes can result in their joining; this mechanism of chromosomal translocation generation largely relies on the spatial proximity of DNA ends. However, whether the potential translocation partners should be initially proximally close when the breaks occur or they can move in the nuclear space after the break to get closer is still an open question. In this regard, the “contact first” and “break first” models of translocation formation have been proposed, which assume that the movement of intact chromatin or the movement of DSBs, respectively, contribute to the eventual proximity of DNA ends and translocation [3,75,91,92].

In the nuclear landscape of mammalian cells, chromosomes are organized into spatially distinct territories where they interact with each other more than with those belonging to the other different territories [93]. The term chromosome territory (CT) was actually first introduced by Boveri in the early 1900s in his studies on the blastomere stages of horse roundworms [94,95]. These CTs are composed of chromatin fibers [96]. Different types of cells and tissues have been observed to have different patterns of CTs in their nuclei [97,98]. The proximity of translocation-prone loci (e.g., *MYC/IGH* or *IGH/BCL6*) is largely determined by the higher order chromosome territory positioning [99]. In contrast to higher eukaryotes, yeasts do not exhibit this manner of organization of their chromosomes [100].

The nuclear genome architecture can be visualized using fluorescence in situ hybridization (FISH) using conventional or high-resolution microscopy, or it can be inferred using chromosome conformation structure (3C) and its spin-offs [101,102]. Using FISH, it was found that the gene loci that are commonly involved in oncogenic chromosomal translocation are already proximal to each other in pre-cancerous cells, supporting the “contact first” model of translocation formation. This was the case of the *ABL-BCR* translocation involving chromosomes 9 and 22 in CML [103], the *PML-RARA* translocation involving chromosomes 15 and 17 in acute promyelocytic leukemia [104], and the translocations of *MYC* in chromosome 8 with either of the three of the immunoglobulin genes: *IGH* in chromosome 14, *IGL* in chromosome 22, and *IGK* in chromosome 2 in Burkitt’s lymphoma [99]. The *MYC* gene has been often found to be close to the *IGH* gene locus compared with the *IGL* and *IGK* gene loci, which may explain why the *MYC-IGH* translocation is observed in around 80% of Burkitt’s lymphoma cases while the remaining *MYC-IGK* and *MYC-IGL* translocations occupy the rest [105]. Interestingly, the specific chromosomal position of the *MYC* gene, but not its sequence, was proven to affect its involvement in translocations, since the replacement with *MYCN* sequence does not affect its role as a translocation partner [106].

With the advent of advanced staining and imaging technologies, the preservation of the overall 3D structure of the nucleus and the 3D imaging of the whole chromosome have become possible. Using the technology of 3D structured illumination microscopy (3D-SIM) with pan-chromosome-specific paints, Sathitruangsak et al. investigated the positioning of chromosomes 4, 9, 11, 14, 16, 18, 19, and 22 in normal lymphocytes and myeloma cells from treatment-naïve patients with monoclonal gammopathy of undetermined significance or multiple myeloma [107]. They calculated the overlap between five chromosomal pairs and found out that the pair of chromosomes 18 and 19, which does not usually engage in chromosomal translocation in multiple myeloma, has up to 50% less overlap compared to the remaining pairs, which are usually involved in translocations, both in normal and malignant nuclei. The chromosomal pair which has less overlap has fewer chances of physical interaction or proximity, which can then negatively affect translocation formation.

FISH methods examine the distance between and the relative position of specific gene loci in the nucleus while Hi-C methods determine the interactions of the gene loci with each other on the genome-wide scale. In this line, Zhang et al. demonstrated the positive correlation between inter-chromosomal contacts and translocation frequency [108]. They built a high resolution Hi-C spatial organization map of G1-arrested mouse pro-B cells and used high-throughput translocation sequencing to detect the translocations after DSB induction by ionizing radiation. They found that in the condition when DNA damage is random (i.e., DSBs happen with the same frequency in different sites), the spatial proximity between loci is the main factor contributing to translocation formation (Figure 3).

To further demonstrate the role of spatial proximity in oncogenic translocations, the publicly available Hi-C datasets on genome-wide interaction maps of a lymphoblastoid cell line (GM06990) and an erythroleukemic cell line (K562) were analyzed along with the collections of 1533 chromosomal translocations from cancer and germline genomes [91]. They observed that the gene loci that are usually involved in oncogenic translocations, such as *MYC-IGH* and *BCR-ABL*, have high Hi-C contact frequencies in normal cells. With these findings, the authors were able to show that the high contact interaction between any two gene loci can influence translocation formation if DSBs are formed in them.

To conclude, the overall nuclear genome architecture has been shown to have a significant influence on the formation of chromosomal translocation by virtue of spatial proximity, which can be analyzed by measuring the physical distance or calculating the contact frequency between potential translocation partners. 

### 3.2. Transcriptional Activity

Transcriptional activity can influence the genome’s vulnerability to DNA damage and its access to the cellular repair machinery. Endogenous DSBs or DSBs caused by DNA replication inhibitors favorably occur in transcriptionally active genomic regions [87,109,110], which could be explained by transcription-induced DNA damage or the collision of transcriptional and replication forks in S phase. Transcriptionally active chromatin is also more vulnerable to DSB formation induced by ionizing radiation [111]. Consistently, it was observed that genetic rearrangements such as chromosomal translocations preferentially occur in coding regions and in actively transcribed genes compared to the intergenic regions and transcriptionally silent genes [88]. Active gene transcription can cause DNA damage in several ways. Transcription produces short DNA–RNA hybrids called R loops that leave the non-template DNA single strand susceptible to mutagenic activities, including DSBs [63,64,112,113,114,115]. In B cells, AID was observed to bind R loops formed at the *MYC* and *IGH* gene loci and to produce DSBs [116,117,118]. Moreover, AID, through its interaction with Spt5, can be recruited to gene promoters occupied by stalled RNA polymerase II, which explains the prevalence of AID-mediated damages within transcription start sites [119,120].

DNA topoisomerase III beta (TOPB3) has been found to relax the negative supercoiled DNA, reducing the transcription-generated R loops and *MYC-IGH* translocation in mammalian cells [121]. Recent studies have shown that other repair factors suppress chromosomal translocations by modulating the R loop formation [115]. In particular, ATM is a known suppressor of the *MYC-IGH* translocation in vivo [122], and it can also prevent the formation of R loops in proliferating cells [123]. BRCA2, which plays a key role in the HR pathway by recruiting Rad51 to the DSBs, has also been found to prevent R loop formation [124]. The R loop suppression activity of BRCA2 could be part of the coordinated roles between BRCA1 and BRCA2 in the HR pathway, or it could be part of the Fanconi anemia pathway to protect the replication fork [115,125].

However, in the study of mutations in different cancers, euchromatin had a lower mutation rate due to transcription-coupled repair, while heterochromatin and nucleosomal DNA were less accessible to repair machinery and had a higher cancer mutation rate [126]. Meanwhile, a relationship between transcriptional activity and chromatin movement was observed in HeLa cells, where inhibition of transcription with actinomycin D abolished increased transcriptional chromatin mobility and configurational changes during early S phase [127]. Moreover, actively transcribed genes are recruited into shared transcription factories, which increases their spatial proximity and promotes translocation in case DSBs occur among them [128]. This was shown for the genes that are usually involved in chromosomal translocations that cause Burkitt’s lymphoma: *MYC*, *IGH, IGL,* and *IGK*, where they share a common translocation factory [129]. The correlation between high gene transcription, their spatial proximity within the nuclear space, and chromosomal translocation appearance have also been observed in diffuse large B-cell lymphoma [130], anaplastic cell lymphoma [131], and prostate cancer [132].

### 3.3. DNA Damage Response

Eukaryotic cells have evolved complex and tightly regulated mechanisms that coordinate the detection, signaling, and repair of DNA damage [133]. These mechanisms are not always efficient, and the formation of chromosomal translocations is one example when the DNA repair machinery incorrectly repairs DSBs. Chromosome translocations are normally found in peripheral blood cells and the non-neoplastic tissues of healthy individuals [134,135,136,137,138,139], which indicates that their formation should not be seen as a pathological cellular event but rather as an inevitable outcome of DNA repair activities. Only when the chromosomal translocation results in molecular and functional consequences that can disrupt the normal physiological processes and then confer selective survival advantage to the cell does it become pathologically relevant.

DNA breaks are repaired either by homologous recombination (HR) or the non-homologous end joining (NHEJ). The HR pathway is mostly used during S phase of the cell cycle. HR uses the homologous template from the unbroken strand to repair the breakage with high fidelity [140,141]. The NHEJ pathway is cell cycle-independent; it ligates the broken ends together and does not require an extended homology template [142,143]. The DNA ends can be either resected during S/G2 stages or suppressed in the G1 stage; this affects the choice of the repair pathway [144]. The resected DNA produces 3’ overhangs coated with Rad51, forming the nucleoprotein filament that is essential in the strand invasion of a homologous template to start the repair [145]. If the homologous template is provided by the sister chromatid, then faithful repair is performed by the HR pathway as opposed to the protected ends in NHEJ [144]. In the absence of a homologous template from a sister chromatid, the resected DNA end can then be repaired by the alternative NHEJ pathway (a-NHEJ) that uses a microhomology of around less than 100 bp, thus requiring limited resection [146]. Additionally, resected DNA ends can be repaired by single-strand annealing (SSA), but this needs annealing of repeats that are more than 100 bp, thus requiring extensive resection [147,148]. Because of the absence of a repair template, the NHEJ pathway results in erroneous repair, ranging from the small indels or mutations at the broken ends up to the relocation of the broken segments leading to chromosomal rearrangements [149]. Whichever pathway the cell utilizes, the repair usually takes from minutes to hours [150]. Both the HR and NHEJ pathways can compete for or even collaborate in DSB repair [151]. DNA repair initiated by the HR pathway can be completed by the NHEJ pathway in some cases [152,153,154], but when it comes to translocation formation, NHEJ is the most proficient, followed by the SSA pathway [149]. 

Translocations result from erroneous DNA repair mediated by one of the three different DNA damage response and repair (DDR) pathways: the classical NHEJ (cNHEJ; mediated by DNA-PKcs and Lig4); alternative NHEJ (aNHEJ; mediated by CtIP, Parp1 and Lig1/Lig3); and the single-strand annealing (SSA; mediated by Rad-52) pathway. Chromosome translocations in rodent and human cells are mainly formed by the alternative NHEJ pathway [155,156,157,158]. SSA-mediated translocations occur predominantly within repetitive DNA elements [159]. The choice of these pathways depends on several factors. 

*Kinetic aspect.* The cNHEJ pathway proceeds very fast (within 2–4 h) [160,161], which makes it the cellular “first-line therapy” of DSBs. Fast kinetics explains the low frequency of translocation formation with cNHEJ [156]: right after the lesion, DSBs are situated close to each other and their direct joining is favored. Consistently, when cNHEJ is slowed down in G1 (a related process known as Artemis- or resection-dependent cNHEJ), the probability of translocation formation is increased [160,162]. An impairment or slow progression of cNHEJ allows aNHEJ to manifest as a backup process [156,161]. The HR pathway proceeds slower than the NHEJ pathway [163] and has a priority over NHEJ in G2 in repairing DSBs situated in heterochromatin regions, complex, or slow-to-repair lesions [163,164,165]. Abrogated or stalled HR can be rescued by the aNHEJ pathway [161].

*Spatial aspect*. Different types of DNA repair have a spatial selectivity as well, which is influenced by the chromatin context. In the study of CRISPR/Cas9-induced DSBs, cNHEJ was found to largely comprise the main DNA repair mechanism both for euchromatin and heterochromatin, while aNHEJ participates in a small proportion of DSBs repair, although its contribution to DSB repair increases in heterochromatin regions [74]. Another study has shown that the DSBs induced by CRISPR/Cas9 in pericentric heterochromatin are immobile and repaired by NHEJ in G1, while in S/G2 they move to the periphery of heterochromatin and are repaired by HR [166]. In addition, the centromeric DSBs that are majorly peripheral recruit both NHEJ and HR factors throughout the cell cycle [166].

*Cell type aspect.* The demand for sister chromatids as repair templates restricts HR to S/G2 phase; thus, NHEJ is considered as the dominant repair pathway in most of the human cells. There are, however, some notable exceptions. DSB repair in human embryonic stem cells is largely dependent on HR due to the prolonged S phase, which facilitates the accurate repair of DNA breaks [167]. The differentiation of human embryonic stem cells decreases DNA repair through HR [168]. In some cases, cell differentiation accompanied by chromatin condensation may even completely suppress DSB repair response [169]. Cancer cells often grow in hypoxic conditions, which may also preferentially inhibit HR and contribute to increased rates of mutation [170].

### 3.4. Chromatin State and Mobility

The DNA in eukaryotic cells is packaged into nucleosomes and then further compacted into denser chromatin [171]. Each nucleosome is composed of around 150 bp DNA that is wrapped around a set of histone octamer [172] containing two copies of H2A, H2B, H3, and H4 [173,174]. The realization of the highly dynamic nature of chromatin has uncovered various regulatory mechanisms that affect different cellular activities such as cell division, DNA replication, DNA repair, and gene expression [175,176]. Chromatin contains epigenetic marks which consist of histone modifications, histone variants, and regions of open chromatin whose distribution and activity are shown to be different in different cell types and diseases [177,178]. The combination of these marks in their spatial context constitutes the chromatin state that encompasses genomic elements such as promoters, enhancers and different regions which are either transcribed, repressed or repetitive [179,180]. Using live cell tracking of chromosome loci tagged with either GFP or fluorescent topoisomerase II, scientists were able to show in yeasts and in Drosophila that the chromatin moves in a suggestive Brownian motion, right into a confined subregion of the nucleus [181]. This constrained motion of chromatin was also observed in bacteria and mammalian cells whose chromatin movement ranges from 10^−4^ to 10^−3^ µm^2^/s [182]. However, chromatin movement does not fully follow the Brownian motion. Chromatin movement has been shown to be dependent on ATP levels and is affected by temperature [183,184,185]. It was also shown that movement of the intact chromatin can be affected by the cell cycle [127,186,187,188,189,190], transcription activity [184,191,192,193], and chromatin condensation [184,194,195]. Physiological chromatin movements occurring within the cell nucleus many times a day can predispose to the chromosomal translocation formation; however, other events should take place to produce translocations. 

The chromatin state was shown to affect the chromatin mobility as chromatin decondensation without changes in transcription activity resulted in the repositioning of genes [195] in a manner similar to that of the transcription-induced chromosomal repositioning [191]. In yeasts, actin-dependent increase of chromatin mobility [194] was observed after targeting the nucleosome remodeling complex INO80 and INO80-dependent nucleosome remodeling [184]. On the other hand, a 30–40% reduction in chromatin density in mammalian cells was observed after DSB induction [196]. In Drosophila, the DSBs located in the heterochromatic region move out from their original domain to be repaired [197]. Specifically, the DSBs in yeasts and mammalian cells were found to move out into the periphery of the heterochromatin [198,199], and this was independent of chromatin state, at least in mammalian cells [166]. However, DSBs associated with the nuclear lamina were observed be repaired differently compared to relocated DSBs [200]. On the other hand, an increase in condensin II activity was found to promote chromatin condensation, spatial separation of CTs, decreased CT contacts and intermixing, and, as a result, decreased translocation frequency after DSBs induction in Drosophila cells [201].

### 3.5. DSB Movement and Clustering

Upon DNA damage, repair factors flock the sites of breakage and form the microscopically-detectable DNA repair foci [202]. The movement of DSBs is usually investigated using time-lapse microscopy on fluorescently tagged DNA repair foci. Upon DSB induction, the resulting chromosomal breaks in yeast and mammals were observed to remain physically close to each other and were positionally stable over time [203,204,205]. 

In yeasts, DSBs were observed to group together in common repair centers [206]. In contrast in mammalian cells, live cell tracking of several DSBs revealed that DSBs did not group together or form clusters, although there were instances when they did, but they were only transient and were observed to be reversible [196,207]. The DNA repair foci after induction of up to four DSBs were also tracked and were found to remain separated [92,205]; they only came together or clustered to a common repair focus if they were to be translocated [92]. The DSBs in mammalian cells were only observed to cluster in common repair foci in case of induction of multiple DSBs (around 100 per cell) [208]. The clustering of multiple DSBs in common repair foci in mammalian cells could be due to the random motion of the breaks, increasing the probability that they meet and group together, or it could be due to the overall control mechanism, as placing the DSBs together in common repair foci can increase the chances that they be illegitimately joined and produce oncogenic translocations [182]. Another possibility is that there are just a limited number of repair foci which the cell can form, thus DSBs are driven to these repair foci similarly to genes recruited into shared transcription factories [128]. The nucleolus is a hub for many nuclear functions [209] formed by rDNA genes located on acrocentric chromosomes. Acrocentric chromosomes are actively involved in chromosomal translocations [210] and several derivative chromosomes remain associated with the nucleolus; this affects expression of proto-oncogenes [31]. Recently, genes whose transcription was inhibited by DSBs were shown to cluster in the vicinity of the nucleolus [211,212]; this potentially increases the probability of translocation, including those with the acrocentric chromosomes.

In mammalian cells, DSBs exhibit similar mobility with that of intact chromatin with a mean displacement of around 1 µm^2^/h [182,196,207]. This includes DSBs formed after exposure to ultrasoft X-rays [213] and DSBs induced by endonucleases [205]. On the other hand, large-scale DNA damage from exposure to α-particles resulted in an extensive movement and clustering of DSB-containing chromosome domains [214]. This was also the case after exposure to γ irradiation, where DSB mobility was observed to be at least twice as high as that of the intact loci [186]. γ Irradiation was shown to induce repositioning of *ABL* and *BCR* genes in the nuclear center of lymphocytes [103]. When breaks were induced by I-SceI endonuclease, the translocating DSBs were observed to exhibit higher mobility compared to the non-translocating DSBs; 5% of the translocating DSBs were capable of traveling long distance (up to 5 µm) [92]. This observation supported the notion that DSBs do search and move to find their respective translocation partner, supporting the “breakage first” model of translocation formation.

In yeasts, breakage induced by I-SceI resulted in the increased mobility of both the induced DSBs and the intact chromatin [215,216,217,218]. The increased DSB mobility in yeasts was shown to be dependent on the homologous recombination factors such as Rad51 [215,217,219,220,221,222] as yeasts do not have other repair pathways than the HR pathway and DSBs need to search the entire nucleus for homology [140].

### 3.6. Cell Cycle

Cell cycle stage determines the state of cellular chromatin and how it is repaired when DSBs occur. As such, it can potentially affect translocation formation. Chromatin mobility has been observed to be different across cell stages, with the highest mobility observed during the early G1 phase and thereafter, the chromosome territories remain quite stable from mid G1 to late G2 [187,189]. During the S phase, the highest mobility can be observed in the early S phase [127]. However in a study where the entire cell cycle phases were considered, no significant differences in chromatin movement were observed between cells in G1, S, and G2 [190]. Nonetheless, the chromatin movement can lead to distancing or approaching of specific gene loci during the cell cycle. Namely, it was shown that the spatial proximity between *ABL-BCR* and *PML-RARA* pairs increases in the period from late S to G2 in hematopoietic precursors and lymphoid cells [104]. The “natural” mobility of intact chromatin does not always reflect the mobility of DSBs that occurs throughout the cell cycle. It was shown that DSBs, induced by ionizing radiation, had decreased mobility during S phase compared with G1/G2 [186]. In contrast, DSBs induced by CRISPR/Cas9 in pericentric heterochromatic regions were shown to be positionally stable in G1 and then move towards the periphery in G2 [166].

How the cell decides which DNA repair pathway to utilize to repair DSBs can be affected by its cell cycle stage: the accurate HR pathway takes place in late S and G2, while the NHEJ pathway is active throughout the cell cycle [140,164]. However in centromeric heterochromatin regions, it was shown that the HR pathway is active both in G1 and S/G2 [166]. Surprisingly, the HR pathway does not have a priority over NHEJ in repairing DSBs during G2 and only accounts for the repair of ~15% of ionizing radiation-induced DSBs [163]. The NHEJ pathway has the highest activity observed in the G1 phase, while the HR pathway has its highest activity in S phase [223]. Although the S/G2 phase requires accurate DNA repair to maintain genomic stability, chromosomal translocations can still be generated at any time as the NHEJ pathway is active all throughout the cell cycle. However, experimentally, no changes in DSB pairing and translocation formation were detected at different cell cycle stages after ISceI-induced DSBs in NIH3T3duo cells [92]. Perhaps this observation could be due to site- and cell-specific conditions, and extrapolating this to the general conclusion about the role of cell cycle stage on translocation formation is still premature.

## 4. Conclusions

Spatial proximity of the potential translation partners primarily affects translocation formation, perhaps largely because chromosomal translocation is a product of a proximity-based DSB misrepair. However, several other factors come into play and influence each other to affect multiple stages in chromosomal translocation formation (Figure 3), highlighting the sheer complexity of this potentially serious and life-threatening occurrence.
